# LMAN1 serves as a cargo receptor for thrombopoietin

**DOI:** 10.1172/jci.insight.175704

**Published:** 2024-12-20

**Authors:** Lesley A. Everett, Zesen Lin, Ann Friedman, Vi T. Tang, Greggory Myers, Ginette Balbin-Cuesta, Richard King, Guojing Zhu, Beth McGee, Rami Khoriaty

**Affiliations:** 1 Department of Ophthalmology and; 2 Department of Molecular and Medical Genetics, Oregon Health and Science University, Portland, Oregon, USA.; 3 Department of Pharmacology,; 4 Department of Internal Medicine,; 5 Department of Molecular and Integrative Physiology,; 6 Department of Cell and Developmental Biology,; 7 Cellular and Molecular Biology Program,; 8 Medical Scientist Training Program,; 9 Life Sciences Institute, and; 10 Rogel Cancer Center, University of Michigan, Ann Arbor, Michigan, USA.

**Keywords:** Hematology, Bone marrow, Mouse models, Platelets

## Abstract

Thrombopoietin (TPO) is a plasma glycoprotein that binds its receptor on megakaryocytes (MKs) and MK progenitors, resulting in enhanced platelet production. The mechanism by which TPO is secreted from hepatocytes remains poorly understood. Lectin mannose-binding 1 (LMAN1) and multiple coagulation factor deficiency 2 (MCFD2) form a complex at the endoplasmic reticulum membrane, recruiting cargo proteins into COPII vesicles for secretion. In this study, we showed that LMAN1-deficient mice (with complete germline LMAN1 deficiency) exhibited mild thrombocytopenia, whereas the platelet count was entirely normal in mice with approximately 7% *Lman1* expression. Surprisingly, mice deleted for *Mcfd2* did not exhibit thrombocytopenia. Analysis of peripheral blood from LMAN1-deficient mice demonstrated normal platelet size and normal morphology of dense and alpha granules. LMAN1-deficient mice exhibited a trend toward reduced MK and MK progenitors in the bone marrow. We next showed that hepatocyte-specific but not hematopoietic *Lman1* deletion results in thrombocytopenia, with plasma TPO level reduced in LMAN1-deficient mice, despite normal *Tpo* mRNA levels in LMAN1-deficient livers. TPO and LMAN1 interacted by coimmunoprecipitation in a heterologous cell line, and TPO accumulated intracellularly in *LMAN1*-deleted cells. Together, these studies verified the hepatocyte as the cell of origin for TPO production in vivo and were consistent with LMAN1 as the endoplasmic reticulum cargo receptor that mediates the efficient secretion of TPO. To our knowledge, TPO is the first example of an LMAN1-dependent cargo that is independent of MCFD2.

## Abstract 2

LMAN1 serves the endoplasmic reticulum cargo receptor that mediates the efficient secretion of TPO. TPO is the first example of an LMAN1-dependent but MCFD2-independent cargo.

## Graphical abstract

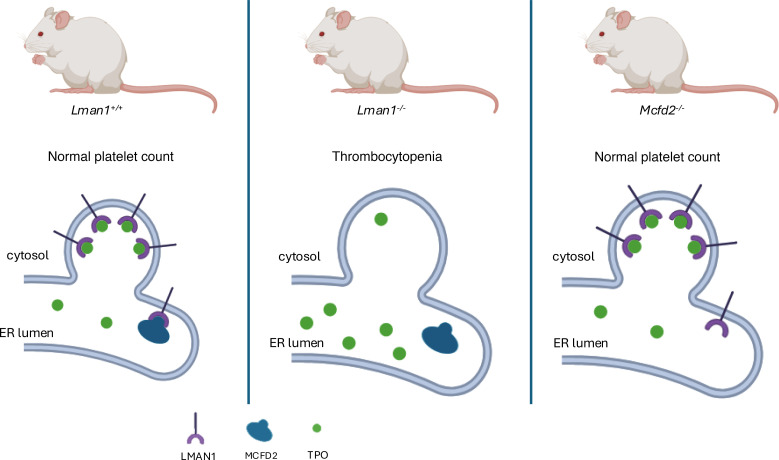

## Introduction

Thrombopoietin (TPO) is a plasma glycoprotein that is produced in hepatocytes and regulates platelet production. Circulating TPO binds to its cell surface receptor, MPL, expressed on megakaryocytes (MKs) and MK progenitors, promoting cell proliferation and maturation and enhancing platelet production (1–7). TPO also increases MK ploidy and expression of lineage-specific surface markers (1, 8) and promotes the formation of the demarcation membrane system (precursor to platelet membrane) and platelet granules (7). Consistent with a critical role of TPO in megakaryopoiesis and platelet production, mice with biallelic germline deletion of *Tpo* exhibit a significant reduction in the number of bone marrow (BM) MKs and peripheral blood platelet counts, both to approximately 10%–15% of normal (9), as well as impaired MK maturity (9). In contrast, mice heterozygous for a *Tpo*-deleted allele exhibit an approximately 40% reduction in BM MKs and platelet counts (9, 10), consistent with a dosage effect between the TPO level and MK/platelet numbers.

In addition to its role in MK development and platelet production, TPO plays a critical role in hematopoietic stem cell (HSC) survival and maintenance (11). TPO-deficient mice exhibit an approximately 70-fold reduction in long-term BM HSCs, though BM cellularity and peripheral blood red blood cell and white blood cell counts remain normal. Mice heterozygous for a *Tpo*-null allele exhibit an intermediate phenotype with an approximately 5-fold reduction in the number of HSCs (11). These and other findings (11, 12) are consistent with a critical role for TPO in HSC maintenance.

In humans, *TPO* mutations that result in enhanced protein translation result in autosomal dominant thrombocytosis (elevated platelet count) (13–18), while loss-of-function mutations in *TPO* (or in its receptor *MPL*) result in congenital amegakaryocytic thrombocytopenia, a disease characterized by thrombocytopenia and absence of BM MKs at birth, with subsequent BM aplasia/failure later in life (19–26). These disorders demonstrate the critical role of TPO in MK and platelet development, as well as in HSC maintenance in humans. Highlighting the role of TPO in platelet production, several TPO-mimetics are FDA approved for treating certain thrombocytopenia disorders and the BM failure disorder aplastic anemia (27–39).

Plasma TPO levels have been shown to be regulated in part by the rate of plasma clearance. TPO binds its receptor MPL on the platelet surface, resulting in its internalization and destruction (40–42). Additionally, in contrast with earlier reports suggesting that *TPO* mRNA is expressed constitutively and at a steady state (9, 43), aged desialylated platelet removal by the Ashwell-Morell receptor (AMR) was found to result in increased hepatic *Tpo* mRNA production (44), and GPIbα expressed on the surface of platelets was shown to induce hepatic *TPO* mRNA production in an AMR-independent mechanism (45, 46). Furthermore, inflammatory states also result in increased hepatic *TPO* mRNA production in vitro and in vivo, an effect mediated by IL-6 (47–51).

Though the transcriptional regulation and plasma clearance of TPO have been well studied, the mechanisms by which TPO is secreted from hepatocytes remains largely unknown. Approximately one-third of the mammalian proteome are secretory proteins (52, 53). These proteins are cotranslationally translocated into the endoplasmic reticulum (ER) and subsequently transported from ER to Golgi via COPII vesicles/tubules before reaching their final destinations (lysosomes, endosomes, plasma membrane, or extracellular space) (54–56). Due to the ER membrane forming a physical barrier between the ER lumen and COPII components, soluble cargoes, such as TPO, either passively flow into COPII vesicles (bulk flow) or are recruited into COPII vesicles by specific cargo receptors or adaptors (cargo capture) (57–62).

To date, lectin mannose-binding 1 (LMAN1) and ER cargo receptor SURF4 are among the few ER cargo receptors that have been well characterized in mammals (63–74). LMAN1, together with its adapter multiple coagulation factor deficiency 2 (MCFD2), form a complex that is required for the efficient secretion of coagulation factors V and VIII and α1-antitrypsin (A1AT) (65, 70–73). SURF4, on the other hand, promotes the efficient secretion of several other cargoes (66–69, 75). Since only a few interactions between soluble cargoes and ER receptors have been described in mammals thus far (57, 62, 76), the extent to which bulk flow versus cargo capture contributes to recruitment of proteins in COPII vesicles is unclear.

We now report that LMAN1-deficient, but not MCFD2-deficient mice, exhibit thrombocytopenia and that mice with combined deficiency of LMAN1 and MCFD2 exhibit thrombocytopenia indistinguishable from that in LMAN1-deficient mice. Tissue-specific deletion of *Lman1* results in thrombocytopenia in mice with hepatocyte-specific *Lman1* deletion but not in mice with deletion of *Lman1* in hematopoietic cells. Plasma TPO level (but not liver *Tpo* mRNA) is reduced in *Lman1*-null mice, with evidence for TPO and LMAN1 physical interaction in heterologous cells in vitro, as well as intracellular accumulation of TPO in LMAN1-deficient cells. Taken together, these results identify TPO as a likely cargo for the ER cargo receptor LMAN1.

## Results

### LMAN1-deficient mice are thrombocytopenic.

**Figure 1:**
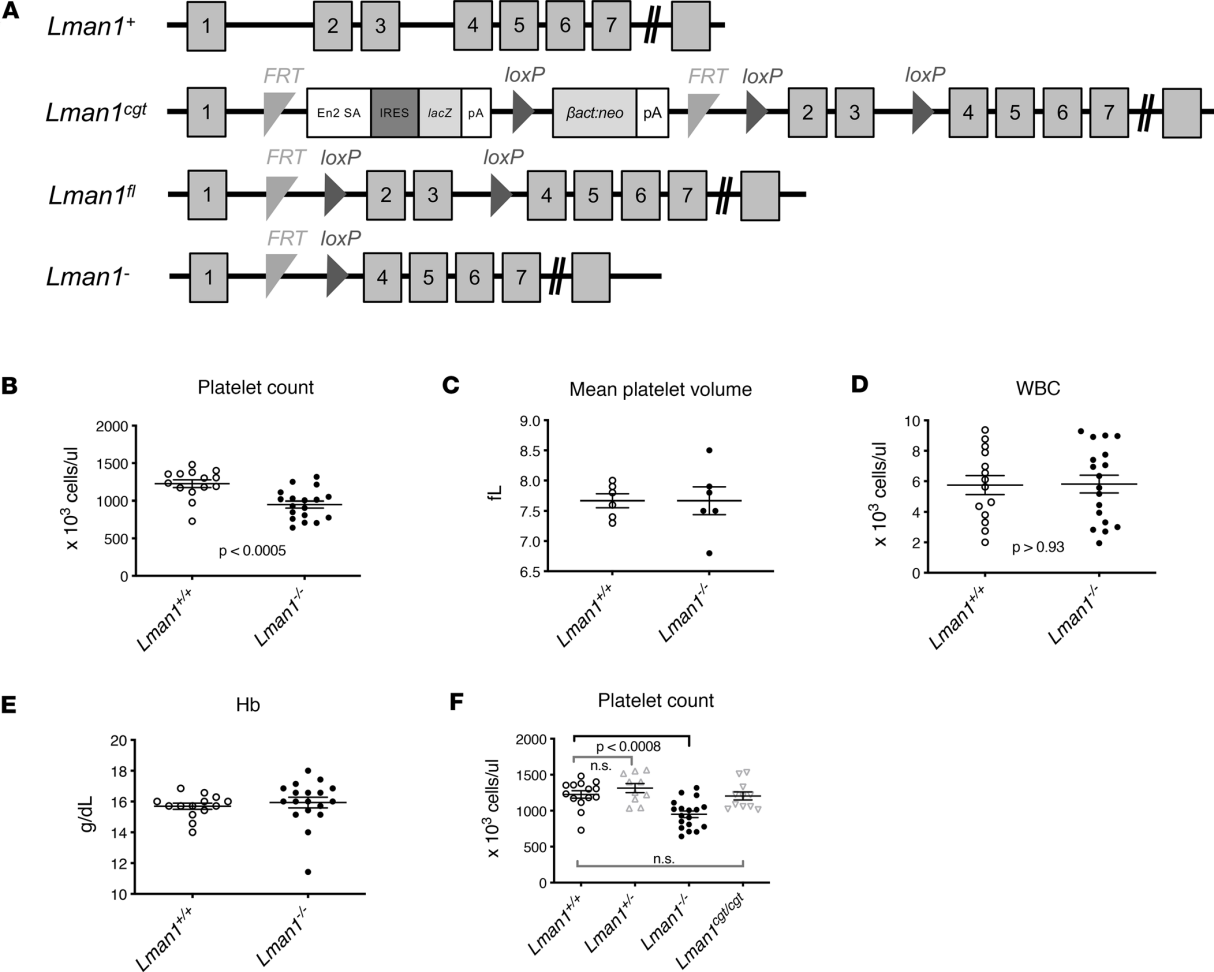
LMAN1-deficient mice exhibit thrombocytopenia. (**A**) The *Lman1* WT allele is denoted *Lman1^+^*. The *Lman1* conditional gene trap allele (*Lman1^cgt^*) allele contains a conditional gene trap insertion in intron 1, which can be excised by expression of FLP recombinase. The *Lman1*-floxed allele (*Lman1^fl^*) is converted to an *Lman1*-null allele (*Lman1^–^*) following Cre-mediated excision of exons 2 and 3. (**B**–**E**) *Lman1^–/–^* mice exhibit (**B**) thrombocytopenia, (**C**) with normal platelet volume, and absence of (**D**) leukopenia or (**E**) anemia. Data were analyzed using unpaired Student’s *t* test. (**F**) Mice with 50% *Lman1* expression (*Lman1^+/–^* mice) or with ~7% *Lman1* expression (*Lman1^cgt/cgt^* mice) exhibit normal platelet counts. Data were compared using 1-way ANOVA, with correction for multiple-comparison testing.

We previously generated mice with a conditional *Lman1* allele (*Lman1^fl^*), in which exons 2 and 3 are flanked by *LoxP* sites (Figure 1A) (77). We crossed the *Lman1^fl^* allele to a mouse expressing Cre-recombinase under the control of the *EIIa* promoter, resulting in germline deletion of exons 2 and 3, a frameshift mutation, and a null *Lman1* allele (*Lman1^–^*) (Figure 1A). In the current study, we examined complete blood counts in samples obtained from *Lman1*-null mice. Surprisingly, *Lman1^–/–^* mice exhibited an approximately 30% reduction in platelet count relative to WT littermates (*P* < 0.0005) (Figure 1B). The mean platelet volume was normal in *Lman1^–/–^* mice (Figure 1C), and no other abnormality on complete blood count analysis was found (Figure 1, D and E).

### Hypomorphic Lman1 mice are not thrombocytopenic.

Mice heterozygous for the *Lman1^–^* allele (*Lman1^+/–^* mice), with 50% *Lman1* expression, exhibited normal platelet counts compared to WT littermate controls (Figure 1F). We previously reported the generation of mice carrying a hypomorphic *Lman1* allele resulting in *Lman1* expression at about 7% of WT levels (*Lman1^cgt^*) (77). To determine if reduced *Lman1* expression to about 7% of normal results in thrombocytopenia, we analyzed complete blood counts in blood samples obtained from the latter mice. We found that *Lman1^cgt/cgt^* mice, which express about 7% of normal *Lman1* levels, exhibited normal platelet counts indistinguishable from WT littermate controls (Figure 1F). These results demonstrate that the thrombocytopenia is only evident with complete LMAN1 deficiency.

### MCFD2-deficient mice do not exhibit thrombocytopenia.

**Figure 2:**
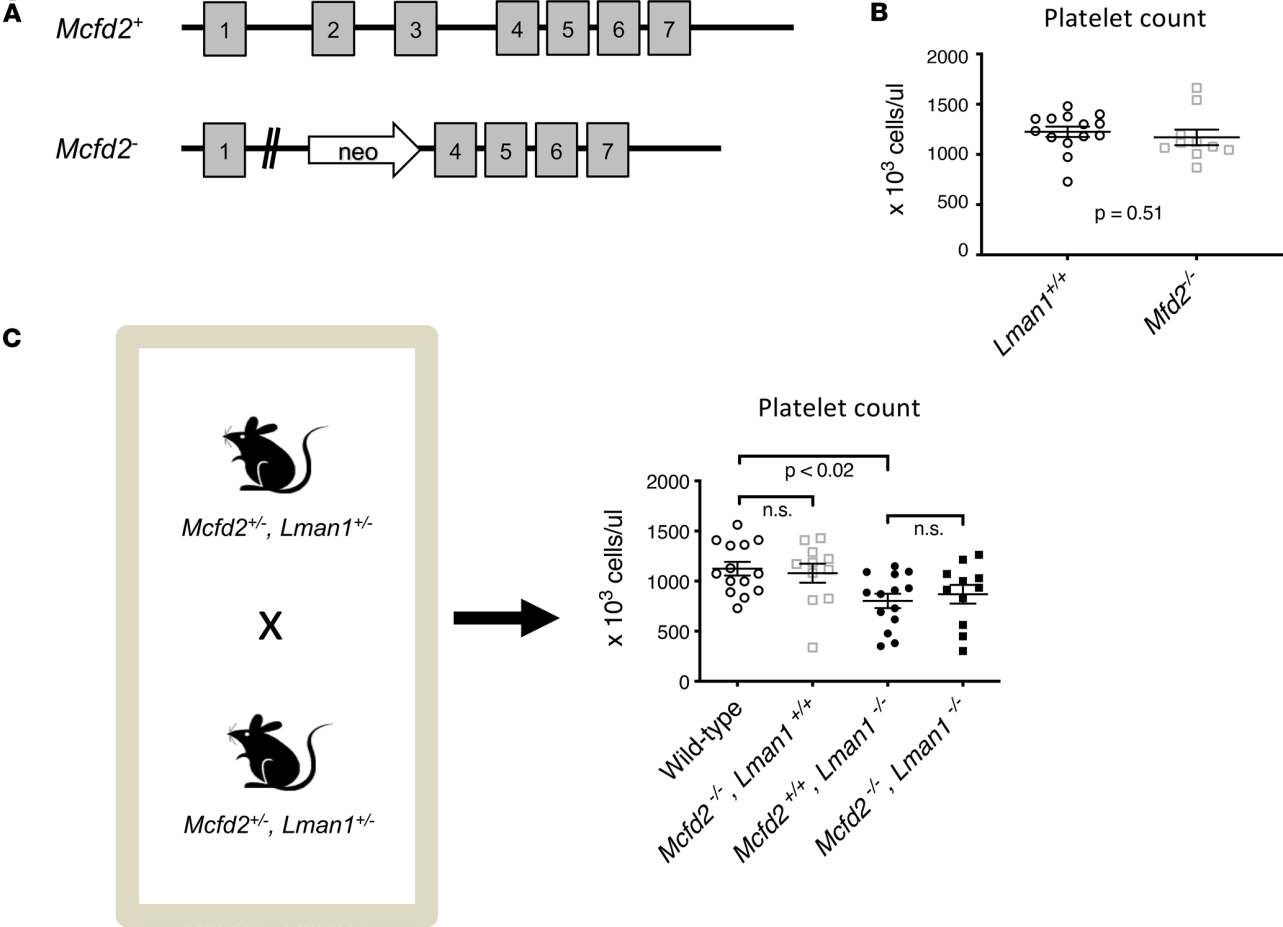
MCFD2 deficiency does not result in thrombocytopenia. (**A**) *Mcfd2^–/–^* mice were evaluated, (**B**) demonstrating normal platelet counts compared to littermate controls. Data were analyzed using unpaired Student’s *t* test. (**C**) LMAN1/MCFD2 double deficient mice exhibit thrombocytopenia, with platelet counts indistinguishable from *Lman1^–/–^* mice. Data were compared using 1-way ANOVA, with correction for multiple-comparison testing.

LMAN1 and MCFD2 form a cargo receptor complex at the ER membrane. Secretory proteins, including factor V, factor VIII, and A1AT, that depend on LMAN1 for secretion, have been shown to also depend on LMAN1’s adaptor MCFD2 for efficient secretion. To determine if MCFD2-deficient mice exhibit thrombocytopenia similar to LMAN1-deficient mice, we generated mice that are homozygous for our previously described *Mcfd2*-deleted allele (*Mcfd2^–/–^*) (78) (Figure 2A). Surprisingly, in contrast with *Lman1^–/–^* mice, we found that *Mcfd2^–/–^* mice exhibited normal platelet counts (Figure 2B).

Next, we intercrossed *Lman1* *Mcfd2* double heterozygous mice to generate mice with combined LMAN1/MCFD2 deficiency. Consistent with the earlier data, analysis of singly deficient mice verified the previously noted mild thrombocytopenia in *Lman1^–/–^* mice (with average platelet count ~70% of WT) with normal platelet counts in MCFD2-deficient mice. LMAN1/MCFD2 double deficient mice exhibited thrombocytopenia, with platelet counts indistinguishable from *Lman1^–/–^* mice (Figure 2C). Taken together, these results suggest that the thrombocytopenia observed in *Lman1^–/–^* mice results from a potentially novel LMAN1-specific (but MCFD2 independent) function, affecting MK/platelet differentiation or survival.

### MK and platelet morphology in LMAN1-deficient mice.

**Figure 3:**
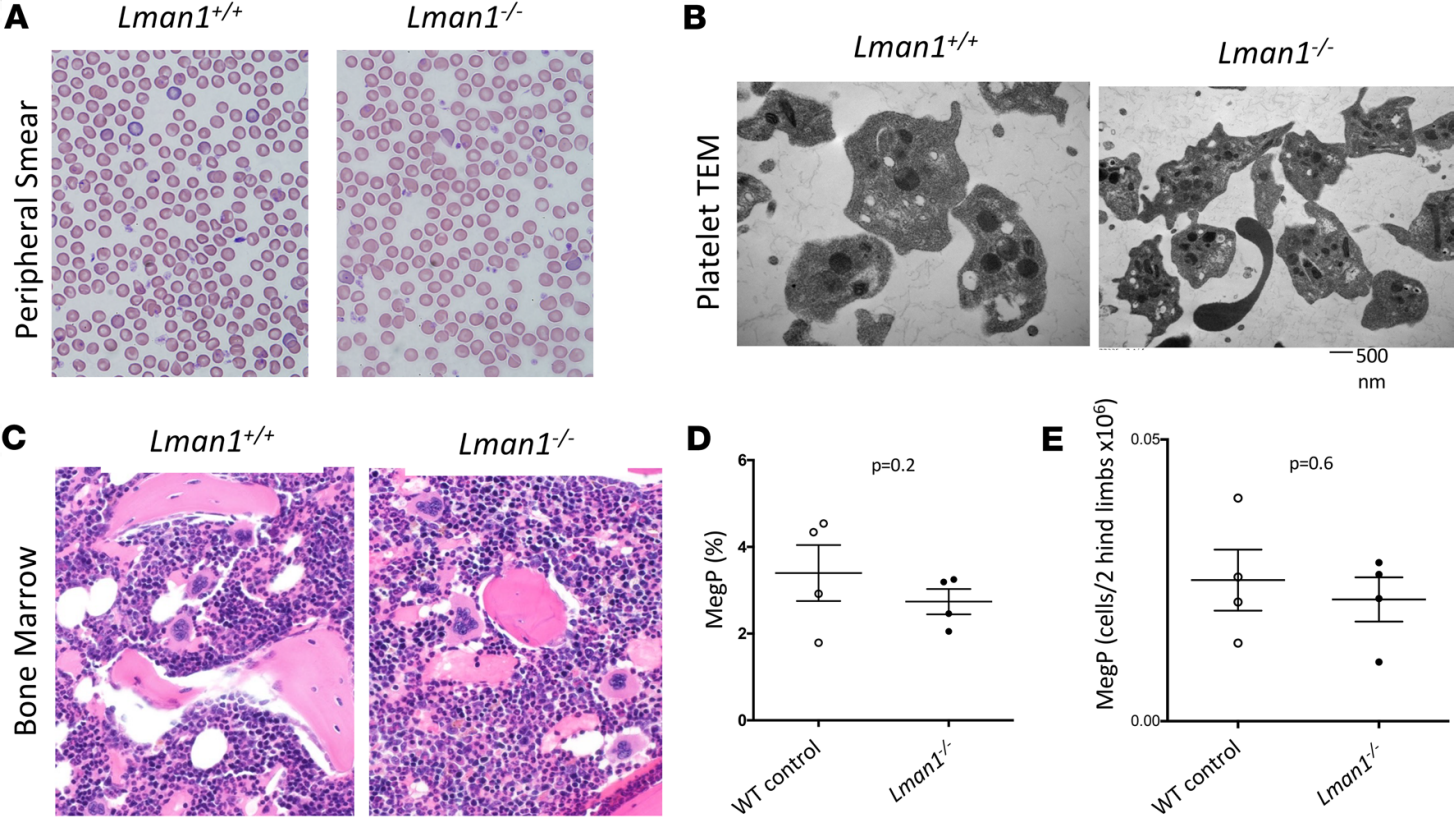
MK and platelet morphology in LMAN1-deficient mice. (**A** and **B**) LMAN1-deficient mice exhibit (**A**) normal platelet size and morphology by peripheral smear evaluation and (**B**) normal platelet dense and alpha granule morphology by transmission electron microscopy (TEM). (**C**) BM histology demonstrates no difference in MK morphology between both genotypes. (**D**) Percentages and (**E**) numbers of BM MK progenitors (Lin^–^Sca^–^KIT^+^CD150^+^CD41^+^) in LMAN1-deficient compared with littermate control mice by flow cytometry. Data were analyzed using unpaired Student’s *t* test.

To define the role of LMAN1 in MK/platelet development or survival, additional studies were performed. Peripheral smears demonstrated normal platelet size and morphology in *Lman1^–/–^* mice (Figure 3A). Next, transmission electron microscopy showed no difference in platelet size or in morphology of dense or alpha granules between *Lman1^–/–^* and WT mice, as evaluated by 2 observers masked to the mouse genotype (Figure 3B). Additionally, histologic examination of *Lman1^–/–^* and WT femurs by 2 observers masked to mouse genotype showed no difference in MK morphology between both genotypes (Figure 3C), but a trend toward reduced number of MKs in *Lman1^–/–^* compared with WT BM was noted (Supplemental Table 1; supplemental material available online with this article; https://doi.org/10.1172/jci.insight.175704DS1). Furthermore, BM analysis by flow cytometry similarly demonstrated a trend (albeit nonsignificant) toward reduced MK progenitors (Lin^–^Sca^–^KIT^+^CD150^+^CD41^+^) in LMAN1-deficient mice (Figure 3, D and E, and Supplemental Figure 1), with no effect on HSCs or early progenitors (Supplemental Figure 2, A–F).

### Lman1 deletion in hepatocytes, but not hematopoietic cells, results in thrombocytopenia.

**Figure 4:**
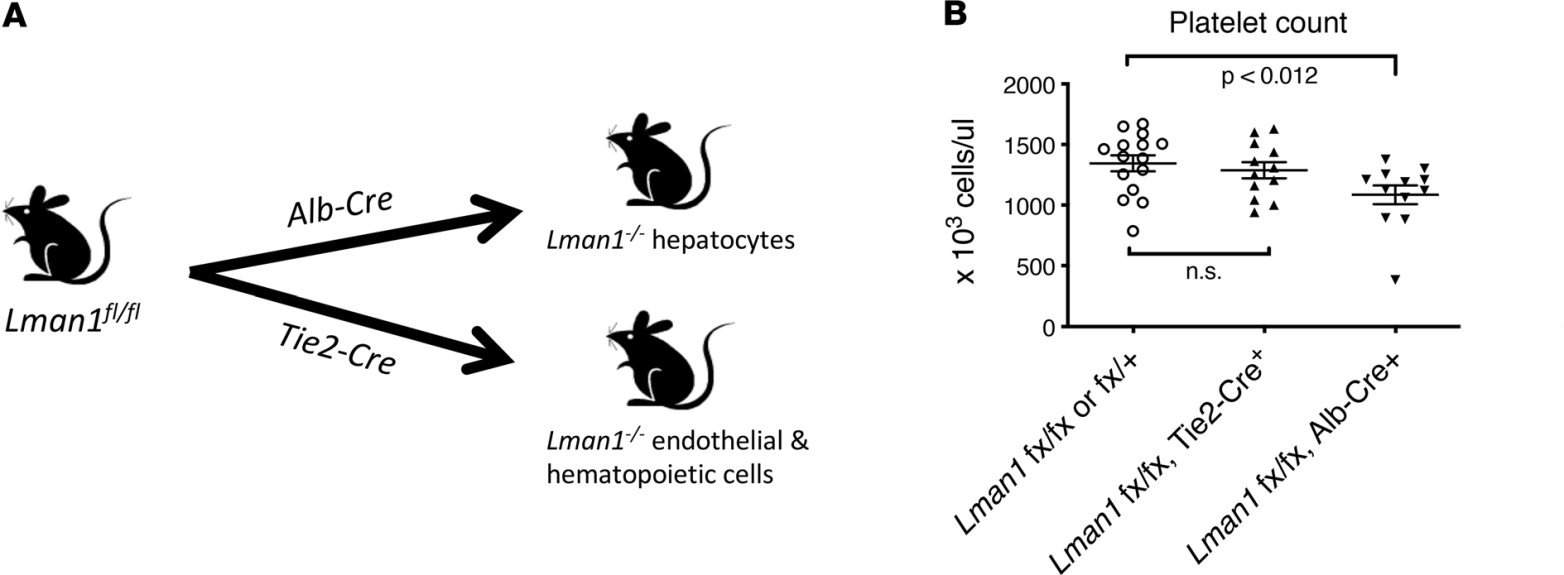
Deletion of *Lman1* in hepatocytes, but not hematopoietic cells, results in thrombocytopenia. (**A**) *Lman1* was deleted in hematopoietic cells using the *Tie2*-Cre transgene and in hepatocytes using the *Alb*-Cre transgene. (**B**) Mice with hematopoietic *Lman1* deletion exhibit normal platelet counts, comparable to those of WT littermate controls. Hepatocyte-specific *Lman1* deletion (using the *Alb*-Cre transgene) result in thrombocytopenia, with platelet counts indistinguishable from those seen in mice with germline *Lman1* deletion. Data were analyzed using 1-way ANOVA, with correction for multiple-comparison testing.

To determine whether the thrombocytopenia results from LMAN1 deficiency specifically in the hematopoietic compartment, mice with tissue-specific knockout of *Lman1* in hematopoietic and endothelial cells were generated by crossing the *Lman1^fl^* allele to the *Tie2*-Cre transgene (Figure 4A and Table 1). To our surprise, platelet counts of mice with hematopoietic LMAN1 deficiency were comparable to those of WT littermate controls (Figure 4B). Thus, the thrombocytopenia observed in *Lman1^–/–^* mice is not due to a defect intrinsic to MKs, platelets, or a hematopoietic cell.

We subsequently generated mice with *Lman1* deletion exclusively in the hepatocytes by crossing the *Lman1^fl^* allele to the *Alb*-Cre transgene (Figure 4A and Table 1). Mice with *Lman1* deletion restricted to hepatocytes exhibited significant thrombocytopenia relative to WT controls (*P* < 0.012) (Figure 4B), with platelet counts indistinguishable from those in ubiquitous *Lman1*-null mice. These data suggest the presence of a potentially novel LMAN1-dependent secretory cargo synthesized in the hepatocyte that contributes to the regulation of platelet count in mice.

### Deletion of Surf4 in hepatocytes does not result in thrombocytopenia.

We additionally generated and analyzed mice with hepatocyte-specific deletion of *Surf4*, an ER cargo receptor that has been shown to regulate the secretion of several mammalian proteins, including proprotein convertase subtilisin/kexin type 9 (PCSK9), erythropoietin, and others (66–69). In contrast with mice with hepatocyte-specific *Lman1* deletion, mice with deletion of *Surf4* in hepatocytes did not exhibit thrombocytopenia (Supplemental Figure 3, A–D), suggesting that SURF4, unlike LMAN1, does not play a role in the secretion of TPO under steady-state conditions.

### Plasma TPO level is reduced in LMAN1-deficient mice.

**Figure 5:**
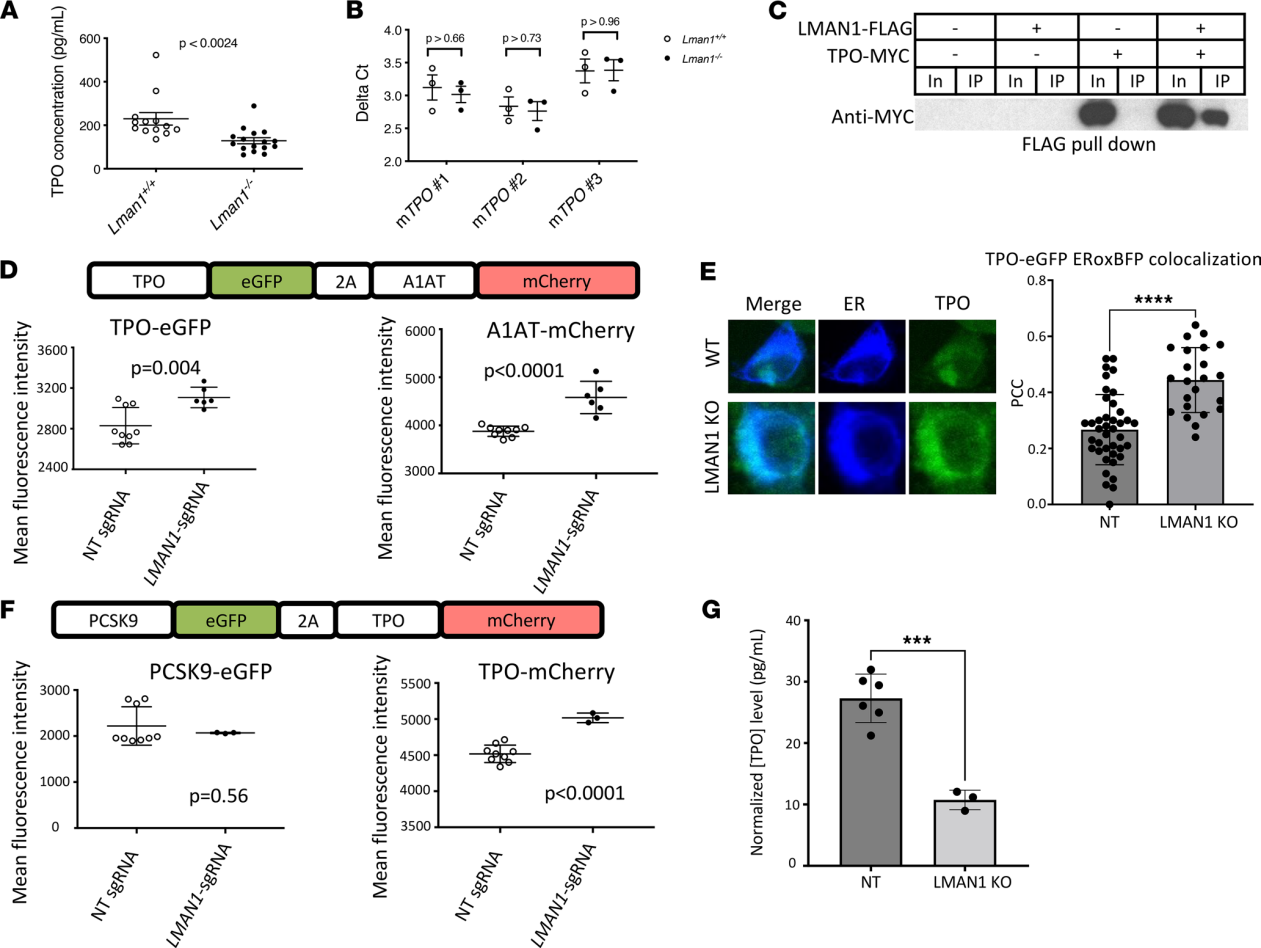
LMAN1 mediates the efficient secretion of TPO. (**A**) Plasma TPO level is reduced in LMAN1-deficient compared with WT littermate control mice. (**B**) *Tpo* mRNA levels were indistinguishable between *Lman1^–/–^* and WT control mice, as demonstrated using 3 different *Tpo* primer sets. (**C**) FLAG-tagged LMAN1 (LMAN1-FLAG) and myc-tagged TPO (TPO-myc) were expressed in HEK293T cells. A physical interaction between TPO and LMAN1 was suggested, as an anti-FLAG antibody coimmunoprecipitated TPO-myc. In, input (10%); IP, immunoprecipitated fraction. (**D** and **E**) A reporter human HEK293T cell line that expresses EGFP-fused TPO and mCherry-fused A1AT was generated. (**D**) Deletion of *LMAN1* using an *LMAN1*-targeting sgRNA resulted in intracellular accumulation of TPO and A1AT compared with cells transduced with a nontargeting (NT) sgRNA. (**E**) Immunofluorescence microscopy demonstrates significantly increased colocalization of TPO in the ER (labeled with blue fluorescent protein) in cells transfected with *LMAN1*-targeting sgRNA (LMAN1 KO) compared with control cells (WT). ****P < 0.0001. PCC, Pearson correlation coefficient. (**F**) A reporter cell line expressing TPO fused to mCherry and PCSK9 fused to EGFP was generated. *LMAN1* deletion results in intracellular accumulation of TPO but not PCSK9. (**G**) *LMAN1* deletion (LMAN1 KO) in HEP3B cells treated with 80 ng/mL IL-6 results in reduced TPO in the supernatant compared with control cells transduced with nontargeting (NT) sgRNA. ***P < 0.001. Statistical analyses in this figure were performed using unpaired Student’s *t* test.

Since TPO is a major hepatocyte-derived regulator of platelet synthesis, we reasoned that TPO production, stability, or secretion could be impaired in *Lman1^–/–^* mice, resulting in thrombocytopenia. Measurement of plasma TPO levels by ELISA demonstrated a reduction in *Lman1^–/–^* mice compared with WT controls (120 × 10^3^ vs. 230 × 10^3^ pg/mL, respectively, *P* < 0.0024) (Figure 5A). However, *Tpo* mRNA levels were indistinguishable between *Lman1^–/–^* and WT livers (Figure 5B). LMAN1-FLAG and TPO-myc coexpressed in HEK293T cells appeared to physically interact, as demonstrated by coimmunoprecipitation of TPO with an anti-FLAG antibody (Figure 5C). In contrast, MCFD2 and TPO did not appear to physically interact by coimmunoprecipitation (Supplemental Figure 4).

Finally, coexpression of TPO fused to EGFP and A1AT fused to mCherry in HEK293T cells demonstrated increased intracellular accumulation of both proteins following deletion of *LMAN1*, consistent with the known dependence of A1AT on LMAN1 for secretion and suggesting a similar dependence for TPO (Figure 5D). Notably, *LMAN1* deletion resulted in significantly increased localization of TPO in the ER (*P* < 0.0001) (Figure 5E). Similarly, a separate reporter cell line expressing TPO fused to mCherry, and as negative control, PCSK9 fused to GFP, demonstrated intracellular accumulation of TPO in *LMAN1*-deleted cells, with no effect on PCSK9 secretion (Figure 5F). Importantly, *LMAN1* deletion in the human hepatocyte cell line HEP3B, which expresses TPO from its endogenous locus, resulted in reduced secreted TPO in the media (Figure 5G). Taken together, these findings demonstrate that LMAN1 is the ER cargo receptor that is required for efficient TPO secretion.

### Lman1-null mice exhibit delayed platelet recovery under hematopoietic stress.

We next investigated if LMAN1 deficiency results in impaired platelet recovery under stress. We exposed *Lman1^–/–^* and WT littermate control mice to chemotherapy (fluorouracil, 5-FU) and measured platelet count recovery over time. Platelet recovery was significantly delayed in *Lman1*-null mice (Supplemental Figure 5).

## Discussion

LMAN1 is a transmembrane protein localized to the ER membrane that, together with MCFD2, forms a receptor complex facilitating the secretion of coagulation factors V and VIII. Loss-of-function mutations in *LMAN1* (or in *MCFD2*) cause the rare autosomal recessive bleeding disorder, combined factor V and VIII deficiency (F5F8D), characterized by reduced plasma levels of these 2 clotting factors to about 10% of normal because of their impaired secretion. The fact that the *LMAN1* gene appeared in evolution before the existence of coagulation factors V and VIII suggests that there are additional LMAN1-dependent cargoes (or other functions for LMAN1); however, to date, only a handful of cargoes have been shown to depend on LMAN1 for secretion.

In this report, we show that: i) mice deficient in LMAN1 exhibit thrombocytopenia; ii) mice with hepatocyte-specific *Lman1* deletion exhibit thrombocytopenia, while mice with hematopoietic *Lman1* deletion do not; iii) plasma TPO level is reduced in *Lman1^–/–^* compared with WT littermate controls; iv) TPO mRNA is unchanged in *Lman1^–/–^* hepatocytes; v) TPO protein accumulates intracellularly in LMAN1-deficient cells; and vi) TPO and LMAN1 physically interact. Collectively, these results, taken together with LMAN1’s known function as an ER cargo receptor, strongly suggest that the thrombocytopenia observed in *Lman1^–/–^* mice is due to impaired secretion of TPO from hepatocytes. The normal platelet volume observed in *Lman1^–/–^* mice is consistent with the generally normal platelet size in patients with congenital thrombocytopenia resulting from a defect in TPO/TPO receptor signaling (79).

In contrast with coagulation factors V and VIII and A1AT (65, 70–73) which require both LMAN1 and MCFD2 for efficient exit from the ER, our results suggest that TPO depends on LMAN1 but not MCFD2 for secretion, the first example to our knowledge of an LMAN1-dependent cargo protein that is independent of MCFD2. TPO could interact with LMAN1 either directly or indirectly via an adaptor other than MCFD2, though no such alternative LMAN1 adaptor proteins have yet been identified. Additionally, while a dose-response relationship between secretion levels of other LMAN1-dependent cargoes and Lman1 expression levels was demonstrated (77), the same does not appear to be the case for TPO.

Of note, patients with combined deficiency for coagulation factors V and VIII have not been reported to exhibit thrombocytopenia. Though the mean platelet count varies widely among different inbred mouse strains, the standard deviation for platelet count measurements within a single inbred mouse strain, such as C57BL/6J, as studied here, is only approximately 10%, affording statistical power to detect subtle difference in platelet count between control and experimental groups even when the sample size is small. In contrast, platelet counts vary considerably among humans and may fluctuate dramatically within the same individual in various settings. Therefore, we hypothesize that the relatively subtle degree of thrombocytopenia detected in our *Lman1*-null mice would likely be missed among the diverse population of human patients with F5F8D, particularly given that a 20%–30% reduction in platelet count is still well within the normal range. It is also possible that the magnitude of the change in TPO secretion and corresponding platelet count reduction is more subtle in humans than in mice. Though we cannot exclude the possibility that TPO is dependent on LMAN1 for efficient secretion in mice but not in humans, our observation that intracellular accumulation of TPO also occurs in a human cell line following deletion of *LMAN1* suggests a similar process in both species.

The primary site of TPO production has been a longstanding controversy. *TPO* mRNA expression has been reported in various tissues, including liver, spleen, kidney, BM, mesenchymal stromal cells, and osteoblasts (2, 11, 80–85). However, the translation of *TPO* mRNA to protein is under stringent control by inhibitory elements in the 5′ untranslated region (86), which limits the cell type(s) that produce the TPO protein. Indeed, recent work using genetically engineered mice that report the expression of TPO at the protein level demonstrated absence of TPO protein expression in most cell types that had been previously implicated in TPO production (except for the liver) (11). Consistent with these results, conditional deletion of *Tpo* from osteoblasts, Lepr^+^ mesenchymal stromal cells, or BM cells resulted in no hematopoietic defects (11). In contrast, hepatocyte-specific *Tpo* deletion (*Alb*-Cre) resulted in hematopoietic defects indistinguishable from those in mice with germline *Tpo* deletion (11). Similarly, *Tpo* deletion in hepatocytes of adult mice also resulted in decreased platelet production (11). These results demonstrate that hepatocytes are the physiological source of TPO both during development and in adult life. Consistent with these findings, liver transplantation for liver cirrhosis results in increased plasma TPO levels 1 day after transplantation, with a subsequent increase in platelet count (87). This report further supports the hepatocyte as the cell of origin for TPO production.

The findings reported here may lay the foundation for the development of new strategies to therapeutically alter plasma TPO level, with potential applications for the treatment of disorders of both low and high platelet counts.

## Methods

### Sex as a biological variable.

Our study included male and female mice, and similar findings are found for both sexes.

### Lman1- and Mcfd2-mutant mice.

Hypomorphic *Lman1^cgt/cgt^* mice with a gene trap insertion in intron 1 of *Lman1* were described previously (88). An *Lman1*-floxed allele (*Lman1^fl^*) in which exons 2 and 3 are flanked by *LoxP* sites was generated by crossing the *Lman1^cgt^* allele to a mouse expressing FLP recombinase from an actin promoter (The Jackson Laboratory stock no. 003800) as previously described (88). Crossing the *Lman1^fl^* allele to mice expressing Cre-recombinase under the control of the EIIa promoter (*EIIa*-Cre, The Jackson Laboratory stock no. 003724) results in germline excision of exons 2 and 3 and a germline null *Lman1* allele (*Lman1^–^*). Mice heterozygous for the *Lman1^–^* allele (*Lman1^+/–^*) were backcrossed to C57BL/6J mice to achieve germline transmission of the *Lman1^–^* allele. The resulting *Lman1^+/–^* mice were intercrossed to generate mice with germline homozygous deletion of *Lman1* (*Lman1^–/–^*). The *Mcfd2*-null allele (*Mcfd2^–^*) with deletion of exons 2 and 3 was also generated as previously described (78). Mice with germline biallelic deletion of *Mcfd2* (*Mcfd2^–/–^*) were generated by intercrossing *Mcfd2^+/–^* mice. All *Lman1* and *Mcfd2* alleles were backcrossed to C57BL/6J mice for more than 8 generations and subsequently maintained on the C57BL/6J genetic background. *Lman1^–/–^* and WT littermate control mice were administered 5-FU (Fresenius Kabi USA, product 101720) at 150 mg/kg body weight once, to induce hematopoietic stress. Platelet counts were measured on days 3, 6, 9, 11, and 15 after chemotherapy administration.

### Tissue-specific Lman1 and Surf4 deletion.

Mice heterozygous for the *Lman1^fl^* allele (*Lman1^+/fl^*) were crossed to mice expressing Cre recombinase under the control of the *Albumin* promoter (*Alb*-Cre^+^ mice) (The Jackson Laboratory stock number 003574) (89). *Lman1^+/fl^ Alb*-Cre^+^ mice generated from the latter cross were subsequently crossed to *Lman1^+/fl^* mice to generate mice with hepatocyte-specific *Lman1* deletion (*Lman1^fl/fl^ Alb*-Cre^+^ mice). Using a similar strategy, mice with deletion of *Lman1* in *Tie2*-expressing endothelial and hematopoietic cells (*Lman1^fl/fl^ Tie2*-Cre^+^ mice) were generated, using the previously reported *Tie2*-Cre allele (The Jackson Laboratory stock number 004128) (90).

Mice heterozygous for a *Surf4*-floxed allele (*Surf4^+/fl^*) were generated as previously described (91), and mice with hepatocyte-specific *Surf4* deletion (*Surf4^fl/fl^ Alb*-Cre^+^ mice) were generated as summarized above.

### Mouse genotyping.

Genomic DNA was extracted from mouse tail biopsies as previously described. Genotyping for the *Lman1^fl^*, *Lman1^–^*, *Lman1^cgt^*, *Mcfd2^–^*, *Surf4^fl^*, *Alb*-Cre, and *Tie2*-Cre alleles was performed as previously described (78, 88, 92).

### Complete blood counts.

Mice were anesthetized briefly with isoflurane, and blood was collected from the retro-orbital venous sinuses as previously described (93). Complete blood count analysis was performed as previously described (94).

### BM histology and flow cytometry.

Femurs from *Lman1^–/–^* and WT control adult mice were harvested to assess BM cellularity and architecture as well as numbers of MKs per BM section. Samples were processed, embedded, sectioned, and stained at the University of Michigan Animal Research Facility. Femurs were fixed in 10% neutral buffered formalin prior to processing, and bones were decalcified in Immunocal (Decal Chemical Corporation) for 24 hours.

Anesthetized mice were euthanized, and BM was flushed from femurs and tibias using RPMI 1640 (MilliporeSigma) supplemented with 5% FBS. BM cells were stained with combinations of the following antibodies: anti-SCA1 (BioLegend 108128 or 108127), anti-cKIT (BioLegend 105826), anti-CD150 (BioLegend 115913 or 115914), anti-CD105 (BioLegend 120403 with secondary antibody staining using BioLegend 405232), anti-CD48 (BioLegend 103404), anti-CD41 (BioLegend 133925), and anti-CD16/32 (BioLegend 101314), as previously described (95). A lineage cocktail consisted of the following antibodies: anti-CD3 (BioLegend 100307 or 100308), anti-CD8 (BioLegend 100708), anti-CD4 (BioLegend 116006), anti-CD11b (BioLegend 101208), anti-CD11c (BioLegend 117308), anti-CD19 (BioLegend 115508 or 557399), anti-B220 (BioLegend 103208), anti–TCR-B (BioLegend 109208), anti–TCR-YD (BioLegend 118108), anti-Gr1 (BioLegend 108408), and anti-NK1.1 (BioLegend 108708). DAPI (MilliporeSigma D8417) or Zombie Aqua Fixable Viability Dye (BioLegend 423102) was used to distinguish dead from live cells. Analysis was performed using FlowJo software (BD Biosciences).

### Electron microscopy.

Platelet-rich plasma and platelet pellets were isolated and processed as follows. A total of 2 mL of room temperature Buffered Saline Glucose Citrate (BSGC) (129 mM NaCl, 13.6 mM Na_3_ citrate, 11.1 mM glucose, 1.6 mM KH_2_PO_4_, 8.6 mM NaH_2_PO_4_, pH 7.3) was placed in a 5 mL polypropylene tube, to which 1–1.5 mL of whole blood was added. BSGC was added to a final volume of 4 mL. The tubes were gently mixed by inversion and were centrifuged at 180*g* for 10 minutes at room temperature without brake. The supernatants (semi-platelet-rich plasma) were removed and centrifuged in fresh tubes at 700*g* for 10 minutes with brake. The resulting isolated platelet pellets were resuspended and prepared for electron microscopy as previously described (94). Briefly, platelet pellets were fixed overnight at 4°C in 0.1 M Sorensen’s buffer (0.1 M Na_2_HPO_4_, 0.1 M KH_2_PO_4_, pH 7.4) containing 2.5% glutaraldehyde. Subsequently, and in this order, platelets were rinsed in 0.1 M Sorensen’s buffer, fixed with 1% osmium tetroxide in 0.1 M Sorensen’s buffer, rinsed in double distilled water, and then stained en bloc with aqueous 3% uranyl acetate for 1 hour. Platelets were dehydrated in ascending concentrations of ethanol, rinsed twice in 100% ethanol, and embedded in epoxy resin. Sample processing and TEM were performed at the University of Michigan Microscopy and Image Analysis Laboratory. Samples were ultrathin-sectioned at 70 nm thickness and stained with uranyl acetate and lead citrate. TEM was performed using a Philips CM100 electron microscope at 60 kV. Images were recorded digitally using a Hamamatsu ORCA-HR digital camera system operated with AMT software (Advanced Microscopy Techniques Corp.).

### Quantitative reverse transcription PCR.

Total mRNA was prepared from livers isolated from adult WT and *Lman1^–/–^* mice, and cDNA synthesis was performed (with on-column DNase I digestion) as previously described (96). Quantitative reverse transcription PCR was performed with SYBR Green RT-PCR Master Mix (Applied Biosystems) using primers listed in Supplemental Table 2 on a 7900HT Fast Real-Time PCR machine (Applied Biosystems). Data were analyzed using the 2^–ΔΔCT^ method as previously described (97), using GAPDH and actin as controls.

### Coimmunoprecipitation.

Mammalian vectors that express FLAG-fused LMAN1 and Myc-fused TPO were generated and transfected in HEK293T cells (ATCC) using Fugene HD transfection reagent (Promega), per manufacturer’s instructions. LMAN1 immunoprecipitation was performed using anti-FLAG antibody covalently bonded to agarose beads (EZview Red Anti-FLAG M2 affinity gel, MilliporeSigma). Immunoblotting with anti-myc antibody (ab10312, Abcam) was performed as previously described (94). To test if MCFD2 physically interacts with TPO, we overexpressed MCFD2-FLAG in HEK293T cells that express TPO-EGFP. At 48 hours after transfection, MCFD2 immunoprecipitation was performed using anti-FLAG M2 magnetic beads (MilliporeSigma, M8823), followed by immunoblotting with anti-EGFP antibody (Abcam, ab290). TPO immunoprecipitation was also performed using chemoTek GFP-Trap Magnetic Agarose (Proteintech, catalog gtma) followed by anti-FLAG immunoblotting (Abcam, catalog ab1238).

### Generation of a TPO reporter cell line.

A construct (CMV-PCSK9-EGFP-p2A-TPO-mCherry) that expresses PCSK9 fused to EGFP and TPO fused to mCherry from the CMV promoter was assembled as previously described (98). HEK293T cells were transfected with this construct using Fugene HD transfection reagent (Promega), and transfected cells were selected with hygromycin (Invitrogen) for 5 weeks. Single cells were subsequently sorted into 96-well plates using SY-3200 flow cytometry (Sony). A clonal cell line that stably expresses PCSK9-GFP and TPO-mCherry was established. Similarly, a construct (CMV-TPO-EGFP-p2A-A1AT-mCherry) was used to generate a clonal cell line that stably expresses TPO-EGFP and A1AT-mCherry.

### LMAN1 deletion in vitro.

An sgRNA targeting *LMAN1* (5′-GATGTGGCAACGCGACCGCG-3′) was generated and cloned into the pLentiCRISPRv2 plasmid (Addgene no. 52961). To prepare lentivirus, pLentiCRISPRv2 was cotransfected with pxPAX2 (Addgene no. 12260) and pCMV-VSV-G (Addgene no. 8454) in a 2:1.5:1 ratio into HEK293T cells at approximately 80% confluence, using Fugene HD transfection reagent (Promega). At 24 hours after transfection, the medium was changed, and viral supernatant was collected 24 hours later. Medium containing viral supernatant was centrifuged at 500*g* for 5 minutes, aliquoted, snap-frozen in liquid nitrogen, and stored at –80°C. pLentiCRISPRv2 lentiviral particles expressing nontargeting control sgRNA were also generated as above. To delete *LMAN1*, cells were transduced with pLentiCRISPRv2 lentivirus expressing an *LMAN1*-targeting sgRNA at a multiplicity of infection of approximately 0.3. Transduced cells were selected with puromycin for 4 days, and analysis was performed about 10 days afterward.

### Live confocal microscopy.

Reporter HEK293T cells that express TPO-EGFP and A1AT-mCherry were transduced with *LMAN1*-targeting sgRNA (listed above) or nontargeting control sgRNA. The latter cells were subsequently transfected with a plasmid expressing ERoxBFP (Addgene no. 68126; ref. 99). At 24 hours after transfection, cells were seeded on Lab-Tek Chambered Coverglass (Thermo Fisher Scientific). Using Nikon IR fluorescence microscope, images were captured. Pearson correlation coefficient was measured using the Nikon NIS-Elements software to analyze the colocalization between TPO and the ER.

### TPO ELISA.

Murine plasma TPO levels were measured by ELISA (MTP00, R&D Systems, Bio-Techne) per manufacturer’s instructions. Secreted TPO was also measured in media of HEP3B cells (ATCC) treated with 80 ng/mL IL-6 (Thermo Fisher Scientific 200-06) for 24 hours, using Human Thrombopoietin Quantikine ELISA kit (Thermo Fisher Scientific DTP00B) per manufacturer’s instructions. Results were normalized to cell counts, assessed by the MTT assay (Roche 11465007001) per manufacturer’s instructions.

### Study approval.

All experiments utilizing mice were performed in accordance with the regulations of the University of Michigan Committee on Use and Care of Animals.

### Statistics.

When 2 groups were compared, data were analyzed using 2-tailed unpaired Student’s *t* test. When 3 or more groups were compared, data were compared using 1-way ANOVA, with correction for multiple-comparison testing. A *P* value less than 0.05 was considered statistically significant.

### Data availability.

*Lman1*- and *Mcfd2*-mutant mice are available at The Jackson Laboratory (068108-JAX and 024426, respectively). Supporting Data Values are included in a supplemental document. All data needed to evaluate the conclusions in the paper are present in paper or the supplement.

## Author contributions

LAE and R Khoriaty conceived the study and designed the experiments. LAE and R Khoriaty performed the majority of the experiments. ZL, AF, VTT, GM, GBC, R King, GZ, and BM performed additional experiments. LAE and R Khoriaty analyzed most of the experimental data. LAE and R Khoriaty wrote the manuscript with help from all authors. All the authors contributed to the integration and discussion of the results.

## Supplementary Material

Supplemental data

Unedited blot and gel images

Supporting data values

## Figures and Tables

**Table 1:**
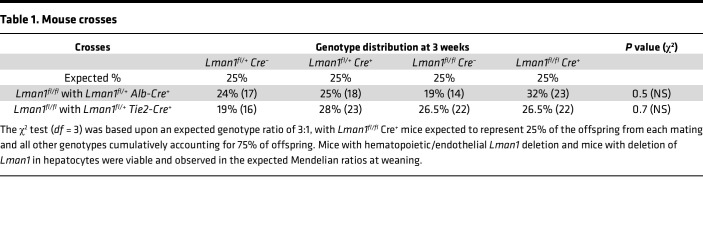
Mouse crosses

## Version 1 11/05/2024

In-Press Preview

## Version 2 12/20/2024

Electronic publication
