# Supplementary material for: LMAN1 serves as a cargo receptor for thrombopoietin
Source: JCI Insight. 2024 Dec 20;9(24):e175704. doi: 10.1172/jci.insight.175704 (PMC11665562; doi:10.1172/jci.insight.175704)
Supplement: Supplemental data [file jciinsight-9-175704-s019.pdf]

Genotype	Average myeloid:erythroid ratio	Average megakaryocyte # / section
Lman1$^{+/+}$	1.79	338.0
Lman1$^{-/-}$	1.77	310.3

Table S1. Number of megakaryocytes in *Lman1*$^{-/-}$ and wildtype littermate bone marrows by histologic evaluation.

Primer	5’ → 3’ Sequence
**qRT-PCR primers**	
Gapdh Fwd	TGTGTCCGTCGTGGATCTGA
Gapdh Rev	ACCACCTTCTTGATGTCATCATACTT
Actin Fwd	CTAAGGCCAACCGTGAAAAG
Actin Rev	GGGGTGTTGAAGGTCTCAAA
m*TPO*1 Fwd	GACTCCCACCTCCTTCACAG
m*TPO*1 Rev	TCCCAGGCTAAAGTCCACAG
m*TPO*2 Fwd	CCTGGGAGAATGGAAAACCC
m*TPO*2 Rev	CTGTCCTCGTGCTGCCA
m*TPO*3 Fwd	ACAGCTTTCTGGGCAGGTT
m*TPO*3 Rev	GGTCCTTGTGAGCTGTGGTC

Table S2. Primer sequences for qRT-PCR.

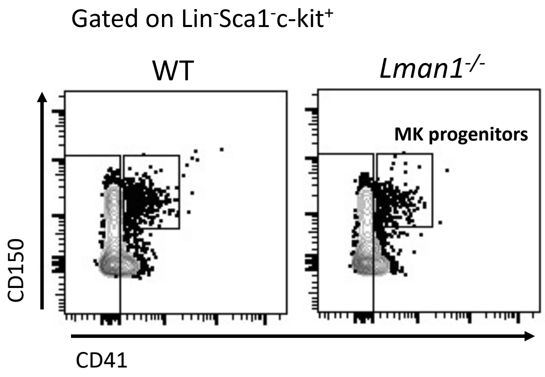

Figure S1. Gating strategy for MK progenitors.

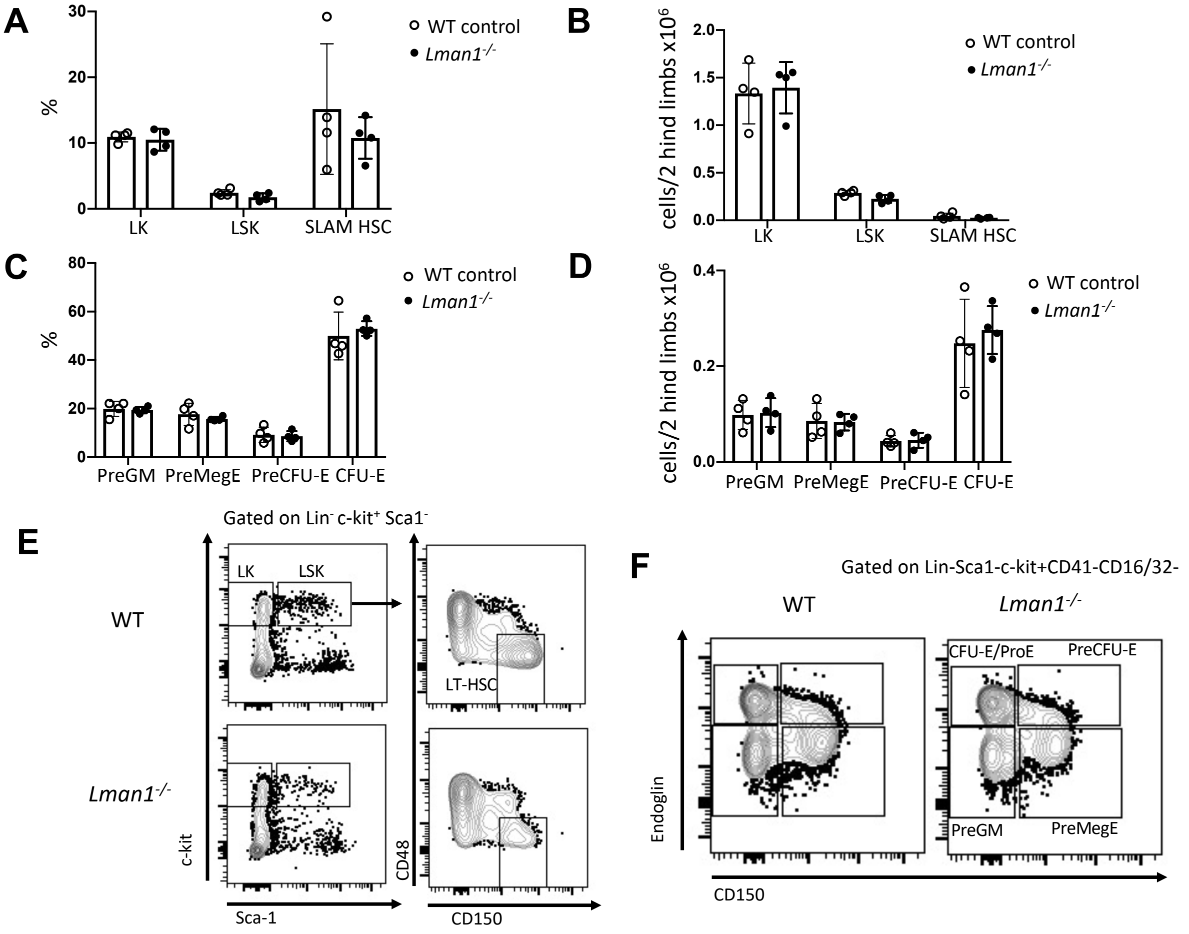

Figure S2. Hematopoietic stem and progenitor cells in *Lman1*$^{-/-}$ and wildtype control mice. LMAN1 deficient mice exhibit normal (A) percentages and (B) numbers of SLAM hematopoietic stem cells (Lin$^-$Sca1$^-$c-KIT$^+$CD150$^+$CD48$^-$), LK (Lin$^-$Sca1$^-$c-KIT$^+$), and LSK (Lin$^-$Sca1$^+$c-KIT$^+$) progenitors, as well as normal (C) percentages and (D) numbers of PreGM (Lin$^-$c-KIT$^+$Sca1$^-$CD41$^-$CD16/32$^-$CD150$^-$CD105$^-$), PreMegE (Lin$^-$c-KIT$^+$Sca1$^-$CD41$^-$CD16/32$^-$CD150$^+$CD105$^-$), PreCFU-E (Lin$^-$c-KIT$^+$Sca1$^-$CD41$^-$CD16/32$^-$CD150$^+$CD105$^+$), and CFU-E (Lin$^-$c-KIT$^+$Sca1$^-$CD41$^-$CD16/32$^-$CD150$^-$CD105$^+$) progenitors. (E, F) Illustration of the gating strategy for LK, LSK, SLAM HSC, preGM, preMegE, preCFU-E, and CFU-E populations.

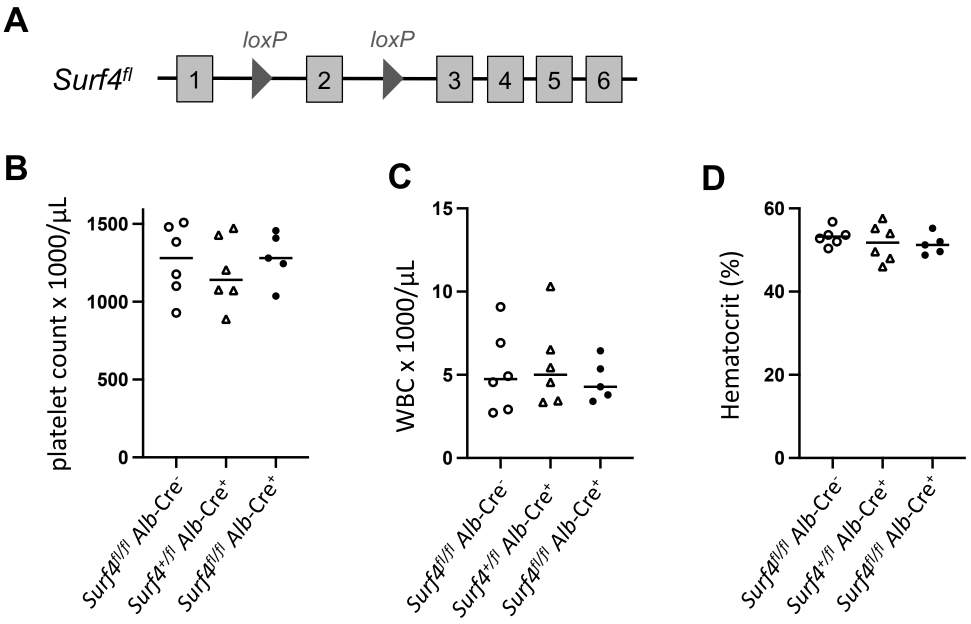

Figure S3. Deletion of *Surf4* in hepatocytes does not result in thrombocytopenia. (A) Mice with hepatocyte-specific *Surf4* deletion were generated. These mice did not exhibit (B) thrombocytopenia compared to littermate controls. *Surf4*$^{fl/fl}$ *Alb*-Cre^{+} mice exhibit (C) white blood cell counts and (D) hematocrit levels indistinguishable from wildtype littermate controls.

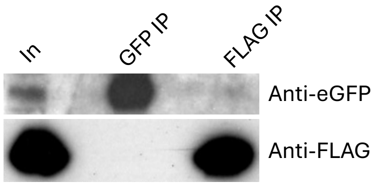

Figure S4. MCFD2 and TPO do not interact by co-immunoprecipitation. FLAG-tagged MCFD2 was expressed in HEK293T cells expressing eGFP-tagged TPO. A physical interaction between TPO and MCFD2 was not apparent, as an anti-FLAG antibody did not immunoprecipitate TPO-eGFP and an anti-eGFP antibody did not immunprecipitate MCFD2-FLAG. IP, immunoprecipitated fraction. In, input (10%).

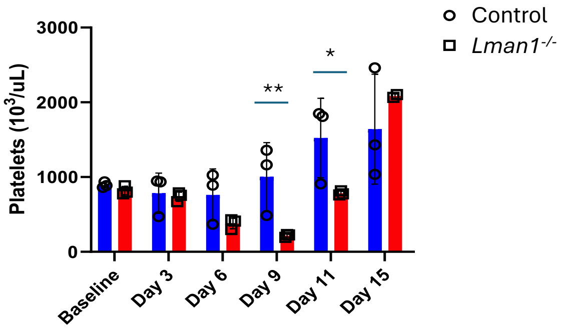

Figure S5. ***Lman1*** **null mice exhibit delayed platelet recovery under hematopoietic stress.** Platelet counts of *Lman1*$^{-/-}$ and wildtype littermate control mice measured on days 3, 6, 9, 11, and 15 following administration of 5-FU. **p<0.01; *p<0.05. Data was analyzed using unpaired Student's t-test.
