# Supplementary figures and images for: LMAN1 serves as a cargo receptor for thrombopoietin
Source: JCI Insight. 2024 Dec 20;9(24):e175704. doi: 10.1172/jci.insight.175704 (PMC11665562; doi:10.1172/jci.insight.175704)

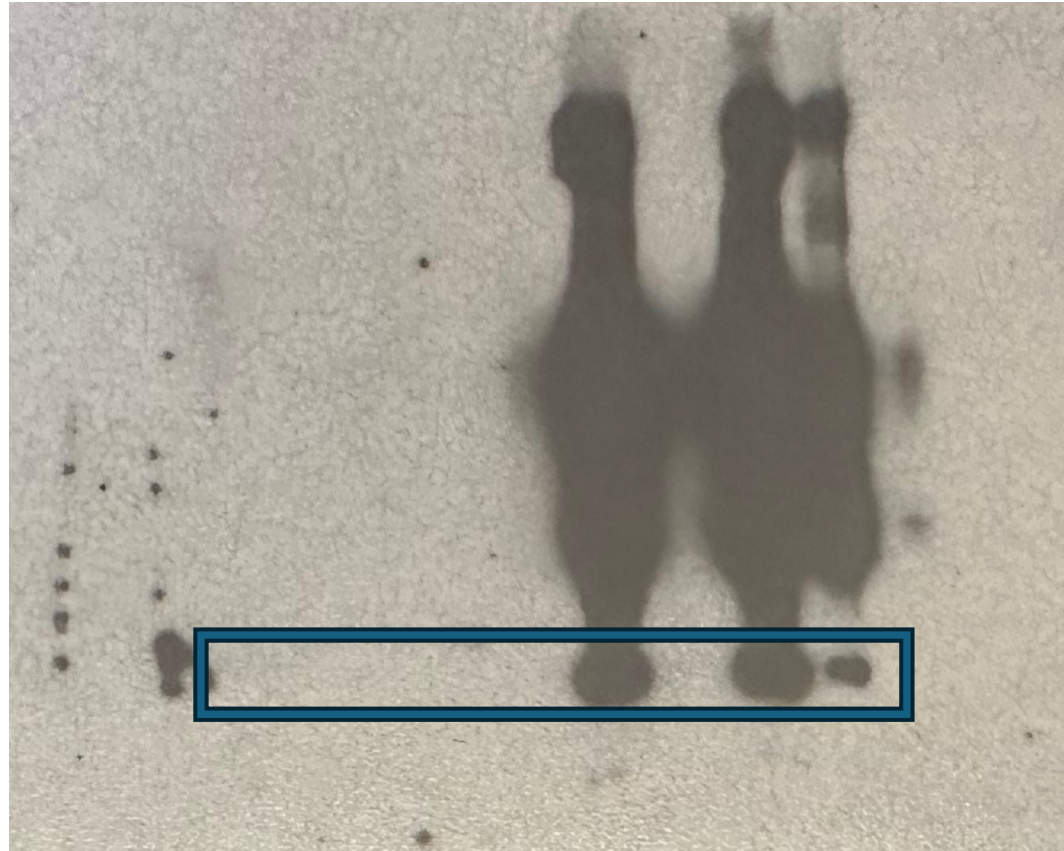

Entire gel for figure 5C

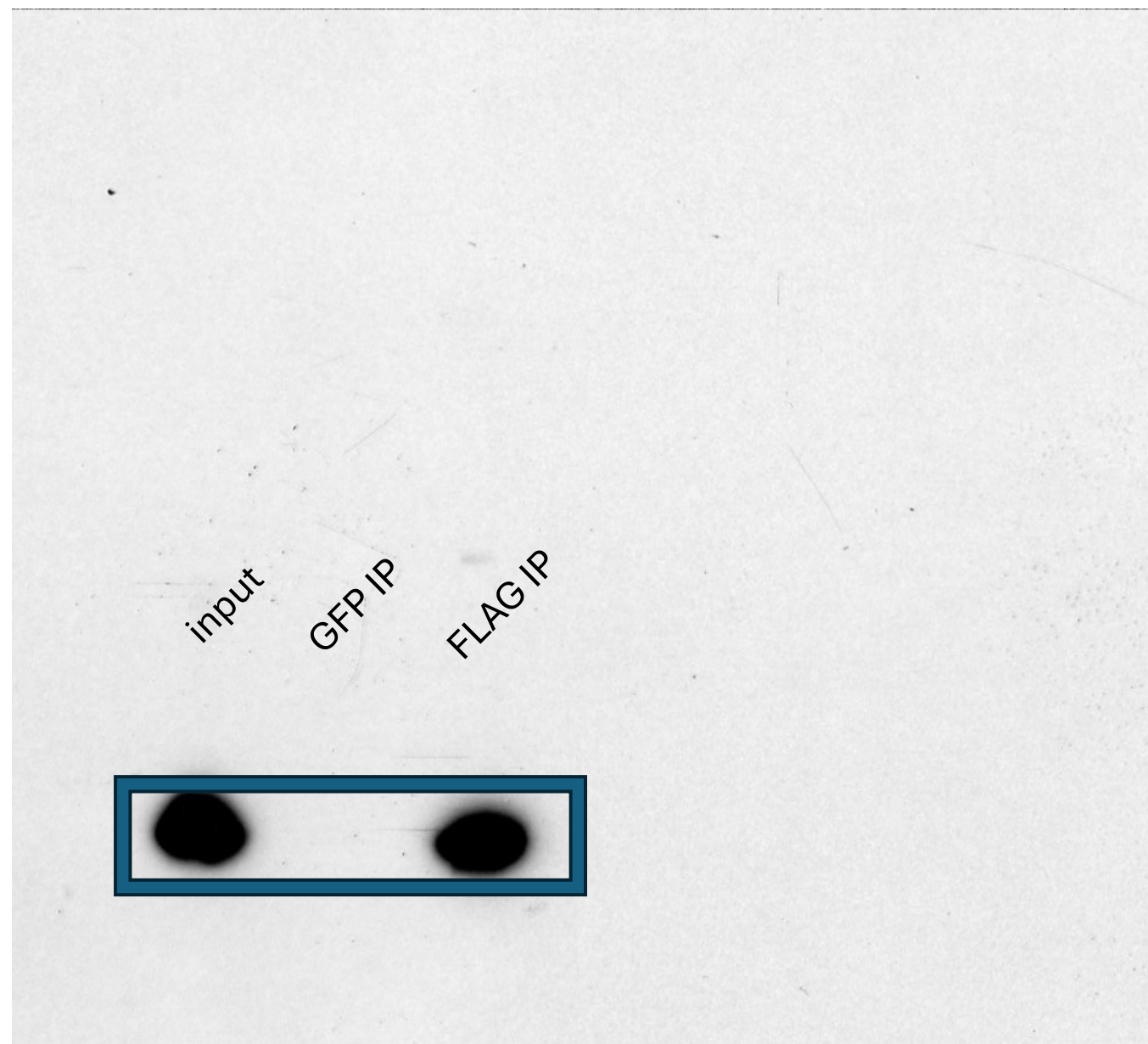

Anti-FLAG whole film for Figure S4

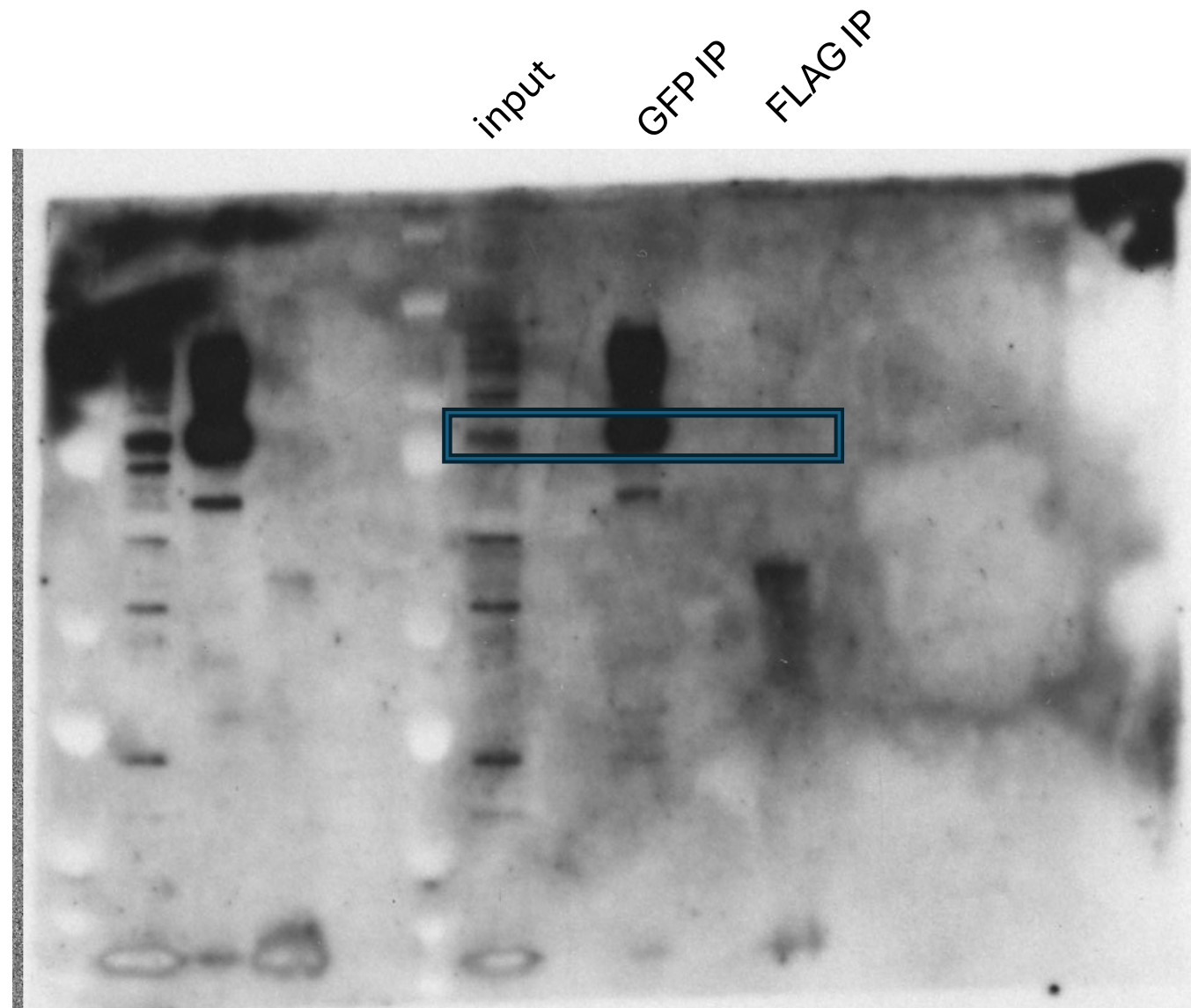

Anti-eGFP whole film for Figure S4

Supplement: Unedited blot and gel images [file jciinsight-9-175704-s020.pdf]
